# B Cell Deficient Mice Are Protected from Biliary Obstruction in the Rotavirus-Induced Mouse Model of Biliary Atresia

**DOI:** 10.1371/journal.pone.0073644

**Published:** 2013-08-21

**Authors:** Amy G. Feldman, Rebecca M. Tucker, Erika K. Fenner, Roberta Pelanda, Cara L. Mack

**Affiliations:** 1 Department of Pediatrics, Section of Pediatric Gastroenterology, Children’s Hospital, Colorado, United States of America, Aurora, Colorado, United States of America; 2 Departments of Medicine and Immunology, University of Colorado School of Medicine, Aurora, Colorado, United States of America; 3 Integrated Department of Immunology, National Jewish Health, Denver, Colorado, United States of America; Baylor College of Medicine, United States of America

## Abstract

A leading theory regarding the pathogenesis of biliary atresia (BA) is that bile duct injury is initiated by a virus infection, followed by an autoimmune response targeting bile ducts. In experimental models of autoimmune diseases, B cells have been shown to play an important role. The aim of this study was to determine the role of B cells in the development of biliary obstruction in the Rhesus rotavirus (RRV)-induced mouse model of BA. Wild-type (WT) and B cell-deficient (Ig-α^-/-^) mice received RRV shortly after birth. Ig-α^-/-^ RRV-infected mice had significantly increased disease-free survival rate compared to WT RRV-infected BA mice (76.8% vs. 17.5%). In stark contrast to the RRV-infected BA mice, the RRV-infected Ig-α^-/-^ mice did not have hyperbilirubinemia or bile duct obstruction. The RRV-infected Ig-α^-/-^ mice had significantly less liver inflammation and Th1 cytokine production compared to RRV-infected WT mice. In addition, Ig-α^-/-^ mice had significantly increased numbers of regulatory T cells (Tregs) at baseline and after RRV infection compared to WT mice. However, depletion of Tregs in Ig-α^-/-^ mice did not induce biliary obstruction, indicating that the expanded Tregs in the Ig-α^-/-^ mice were not the sole reason for protection from disease. ***Conclusion***: B cell deficient Ig-α^-/-^ mice are protected from biliary obstruction in the RRV-induced mouse model of BA, indicating a primary role of B cells in mediating disease pathology. The mechanism of protection may involve lack of B cell antigen presentation, which impairs T-cell activation and Th1 inflammation. Immune modulators that inhibit B cell function may be a new strategy for treatment of BA.

## Introduction

Biliary atresia (BA) is the leading cause of neonatal cholestasis, occurring in approximately 1 out of 10,000 live births in the United States [1]. BA entails a progressive, inflammatory injury of bile ducts, leading to fibrosis and obliteration of both the extrahepatic and intrahepatic bile ducts [2,3]. At the time of diagnosis, a Kasai portoenterostomy is performed in an attempt to re-establish bile flow. Despite this surgical intervention, the intrahepatic bile duct injury continues, leading to cirrhosis and the need for liver transplantation during childhood in the majority of patients [4,5]. A better understanding of the immune mechanisms associated with BA has the potential to lead to new therapies aimed at halting injury to the intrahepatic bile ducts and preserving liver function.

Multiple theories have been proposed as to the pathogenesis of BA, including viral infection [6–8], autoimmune mediated bile duct destruction [2,9] and abnormalities in bile duct development [10]. It is hypothesized that an initial virus infection induces an autoreactive T cell-mediated injury to bile duct epithelium which persists even after virus is cleared [2]. In order to perform mechanistic studies, the Rhesus rotavirus (RRV)-induced mouse model of BA has been employed by many investigators [11–14]. In this model, the bile duct injury is associated with Th1-mediated inflammation and specifically with bile duct epithelial autoreactive T cells [15,16]. Much less is known about the role of B cells in the pathogenesis of BA. Many experimental models of autoimmune diseases have demonstrated an important role of B cells in disease pathogenesis [17–20] and trials of B-cell modulating agents are being conducted in human autoimmune diseases [21]. In both humans and in the mouse model of BA, periductal immunoglobulin deposits and circulating autoantibodies have been described [15,22]. One such autoantibody reactive to cytosolic enolase from bile duct epithelia has been identified in both mouse and human BA [23], as well as in other autoimmune biliary diseases [23,24], lending further evidence to the role of autoimmunity in the pathogenesis of BA.

In this study, we explored the role of B cells in the development of bile duct injury and obstruction in the mouse model of BA through the use of mb-1/CD79A gene knockout (Ig-α^-/-^) mice [25]. These mice have loss of B cell receptor expression and function and are unable to present antigen or produce immunoglobulin [25–27]. In some autoimmune disease models, B cells have been shown to play an important role as antigen presenting cells that activate T cells [18,20]. Therefore, a sub aim of this study was to determine the effect of B cells on CD4^+^ Th1 cytokine response, as a marker of T cell activation, in this model.

## Methods

### Mice

All animals were housed and handled in accordance with criteria outlined in the NIH "Guide for Care and Use of Laboratory Animals" (publication #86-23 revised 1985) through the UC Denver Office of Laboratory Animal Medicine. The study was approved by the University of Colorado Institutional Animal Care and Use Committee. All efforts were made to ameliorate animal suffering. Animal sacrifice was performed by CO2 asphyxiation followed by decapitation for neonates and cervical dislocation for adult mice. Timed pregnant female BALB/c mice were purchased from rotavirus-free colonies of Jackson Laboratory (Bar Harbor, ME). Ig-α^-/-^ mice [25] on the BALB/c background were a gift from Roberta Pelanda, PhD (National Jewish Health, Denver, CO). Mice were given a single intraperitoneal (i.p.) injection of RRV (1.5 X 10^6^ pfu/mL) or balanced salt solution (BSS) within 24 hours of life. Animals were assessed each morning for jaundice in non-fur bearing regions. If animals were noted to display pain, severe distress, suffering, or impending death, they were euthanized immediately. Surviving animals were sacrificed at day 14 and pooled tissues from 3–8 mice were analyzed (minimum 3 pools/ experiment). For experiments involving Treg depletion, Ig-α^-/-^ mice were also given i.p. PC61 (gift from Ron Gill, PhD, Department of Transplant Immunology, University of Colorado, Aurora, CO) or rat serum control. All efforts were made to minimize animal suffering. Animal sacrifice was performed by CO_2_ asphyxiation followed by decapitation for neonates and cervical dislocation for adult mice.

### Tissue histology

Tissue was formalin fixed, paraffin embedded, and stained with hematoxylin-eosin. Digital photographs were obtained using the Olympus BX41 microscopes (Melville, NY).

### Infectious Plaque Assay

Liver tissue was homogenized in 100% w/v BSS and virus concentration was determined by plaque assay as previously described [13].

### Immunohistochemistry

Liver tissue sections (7µm thick) were stained with FITC-conjugated antibodies to CD3 or CD11b (eBiosciences, San Diego, CA) and Alexafluor 555-conjugated cytokeratin 19 using standard protocols [28].

### Serum direct bilirubin and immunoglobulin

Serum direct bilirubin levels were determined with the Direct Bilirubin Test (Diagnostic Chemicals Ltd., Charlottetown, Canada). Serum IgG and IgM levels were determined *via* ELISA according to manufacturer’s instructions (Kirkegaard & Perry Laboratories, Gaithersburg, MD) (Pooled sera from 3 separate experiments).

### Isolation of Immune Cells from Tissue and Flow Cytometric Analysis

Tissue was homogenized and red cells lysed with ACK buffer. Liver immune cells were enriched by Percoll gradient (40/60). Single-cell suspensions were incubated with Fc-block and stained with the following fluorochrome-conjugated antibodies (eBioscience, San Diego, CA): CD45, CD3, CD4, CD8, CD11B, B220, IgM, CD19, NKG2D, Foxp3, CD25, CD11C, or isotype matched controls. A mouse regulatory T cell (Treg) staining kit was used according to the manufacturer’s instructions (eBioscience, San Diego, CA). Cells were visualized with FACS Caliber flow cytometer (Becton-Dickinson, Mountain View, CA) using FlowJo (Tree Star, Inc., Ashland, OR) software for analysis.

### Intracellular cytokine analysis by flow cytometry

Hepatic immune cells were incubated with Brefeldin A. For some experiments, cells were stimulated with phorbol 12-myristate 13-acetate (PMA) and ionomycin. All cells were incubated with fluorochrome-conjugated antibodies (eBioscience, San Diego, CA, USA): CD45.2, CD4, CD8, CD11B, or CD25 followed by permeabilization and intracellular staining for either: IL2, IL17, TNFα, IL10, or IFNγ.

### Statistical analysis

Values expressed as mean±standard deviation. One way analysis of variance (ANOVA) and Bonferroni’s correction were used when more than two groups of mice were compared. The t test was used for comparison between two groups. PRISM Graph Pad software (La Jolla, CA, USA) was employed for statistical analysis and creation of Kaplan-Meier curves. Differences in means were considered significant for p values <0.05.

## Results

### Characterization of B cell knockout status in Ig-α^-/-^ mice.

**Figure 1:**
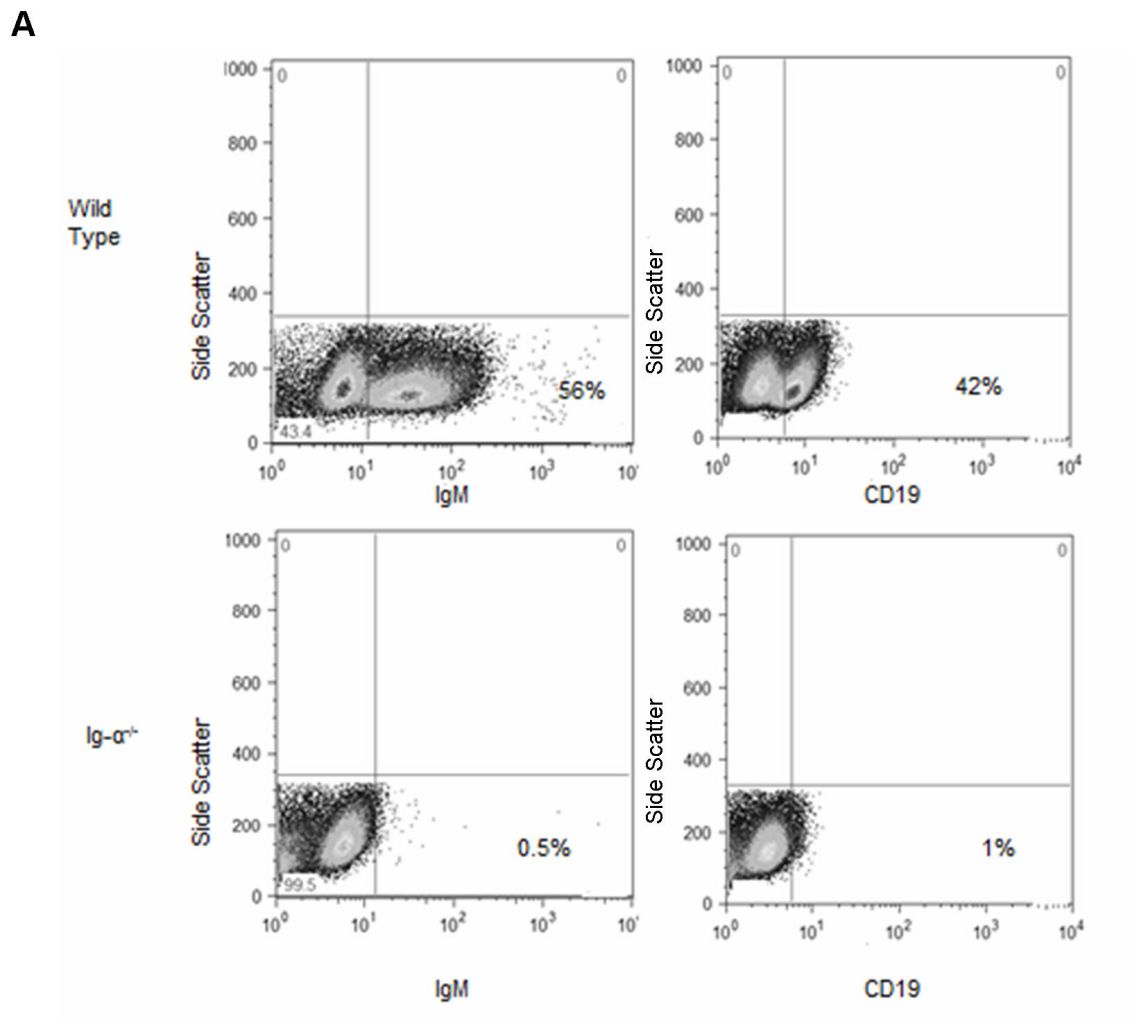
Characterization of Ig-α^-/-^ mice. Representative dot plots of B cell surface marker expression on splenocytes from WT and Ig-α^-/-^ mice, confirming lack of B cells in Ig-α^-/-^ mice.

 The B cell receptor (BCR) is composed of membrane-bound Ig (that binds antigen) and the non-covalently associated signal transduction moiety Ig-α/Ig-β (that is necessary for B cell activation). The BCR is expressed on the cell surface and is functional only when all components are present. The B cell deficient state of the Ig-α^-/-^ mice was confirmed by the lack of cells expressing CD19 and IgM (Figure 1) and by lack of serum IgM and IgG (data not shown).

### RRV infected Ig-α^-/-^ mice are protected from developing BA

**Figure 2:**
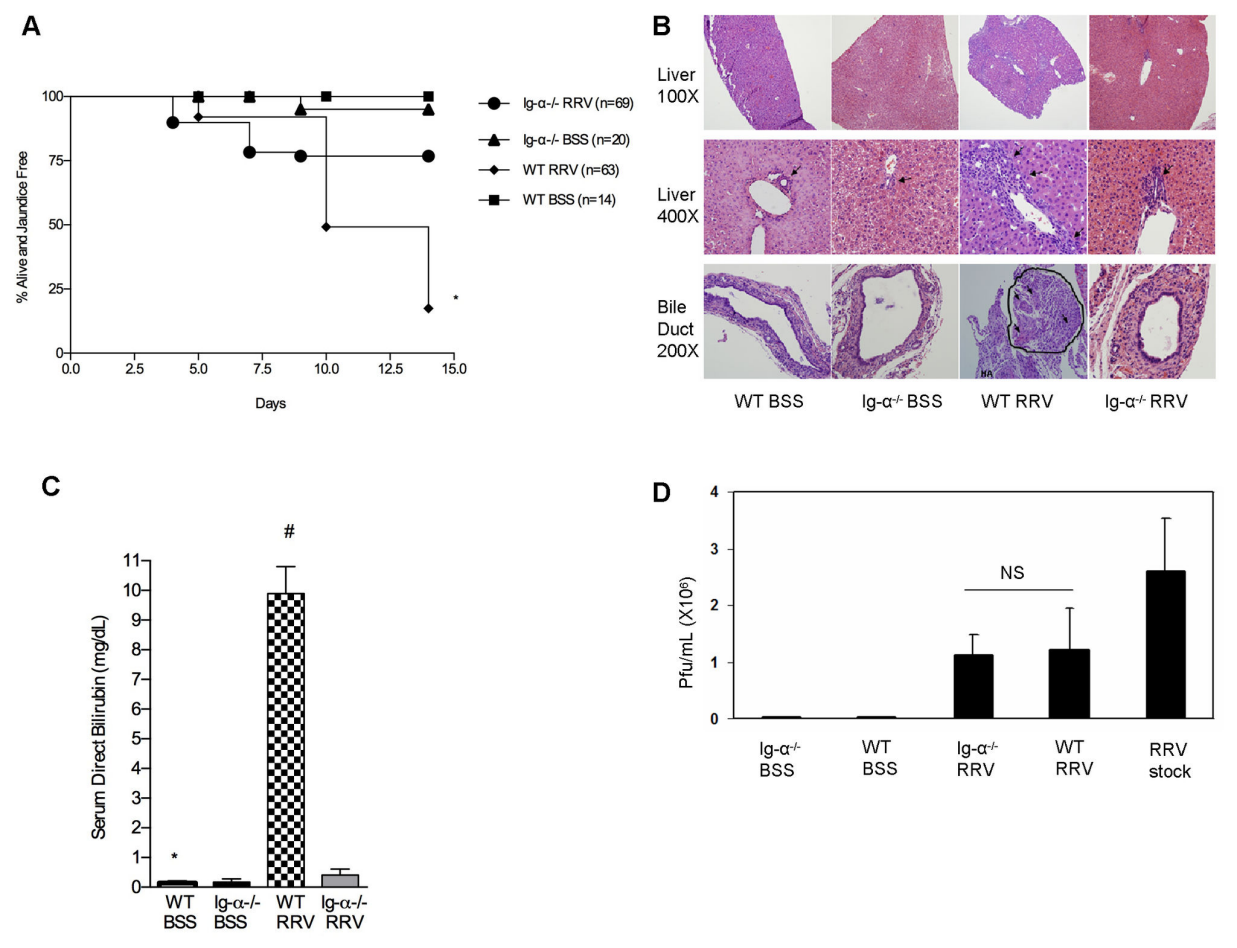
RRV-infected Ig-α^-/-^ mice do not develop bile duct inflammation and obstruction. (A) Disease free survival. Biliary disease was identified based on jaundice and acholic stools. *P<.001 vs. Ig-α^-/-^ RRV mice. (B) Histology. H&E staining from liver and extrahepatic bile ducts (arrows denote bile duct epithelia). 100x: The WT RRV liver inflammation extends between portal tracts. 200x: The WT RRV bile duct obstruction is not seen in the Ig-α^-/-^ RRV mice (HA: hepatic artery). (C) Serum direct bilirubin. *P<.001 vs. WT RRV; #P < .001 vs. Ig-α^-/-^ RRV. (D) Infectious plaque assay. Quantification of infectious virus from 1 week old liver homogenates (mean±SD pfu/ml).

Significantly improved disease-free survival rate was observed at 2 weeks of age in Ig-α^-/-^ RRV-infected mice (76.8%; n=69) compared to WT RRV-infected mice (17.5%; n=63) (P<0.0001) (Figure 2A). The WT RRV-infected mice displayed extensive portal tract and extrahepatic bile duct inflammation and obstruction, a finding not seen in the Ig-α^-/-^ RRV-infected mice (Figure 2B). Serum direct bilirubin levels were significantly lower in Ig-α^-/-^ RRV-infected mice at 2 weeks of age (RRV-infected WT: 10.05±3.09 mg/dL; RRV-infected Ig-α^-/^: 0.41±0.49 mg/dL) (Figure 2C). To determine if RRV infection of the liver was altered in the Ig-α^-/-^ mice, infectious plaque assays were performed. At 1 week, WT and Ig-α^-/-^ mice had similar levels of infectious virus and by 2 weeks both groups had undetectable virus (Figure 2D). These data suggest that B cell deficient mice were protected from the inflammatory-mediated biliary injury and obstruction associated with BA.

### RRV-infected Ig-α ^-/-^ mice have significantly decreased liver inflammatory cells and increased regulatory T cells

**Figure 3:**
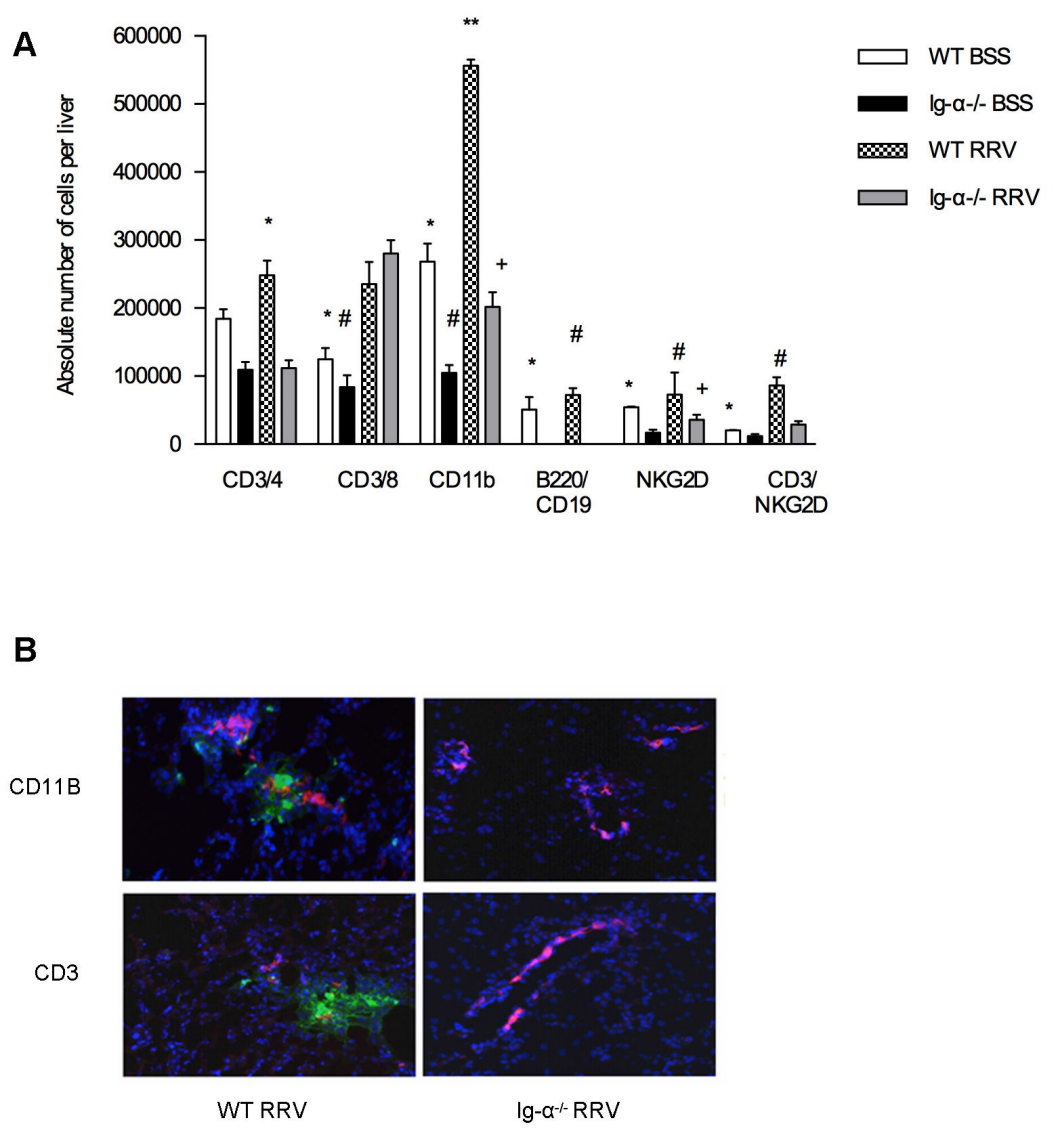
Livers of RRV infected Ig-α ^-/-^ mice have decreased pro-inflammatory cells. (A) Liver immune cell profile in 2 week old mice. Immune cells were identified based on CD45 expression. Shown is the mean±SD absolute number of each immune cell subset per liver. CD3/4: * P<.01 vs. Ig-α-/- RRV; CD3/8: * P<.05 vs. WT RRV; #P<.01 vs. Ig-α-/- RRV; CD11b: * P<.001 vs. Ig-α-/- BSS; ** P<.001vs. WT BSS; #P < .05 vs. Ig-α-/- RRV; + P<.001 vs. WT RRV; B220: *P<.05 vs. Ig-α^-/-^ BSS; #P < .01 vs. Ig-α^-/-^ RRV; NKG2D: *P<.05 vs. Ig-α^-/-^ BSS; #P < .05 vs. WT BSS; +P < .01 vs. WT RRV; CD3/NKG2D: *P<.001 vs. WT RRV; #P < .001 vs. Ig-α^-/-^ RRV. (B) Liver immunohistochemistry. Liver tissue was incubated with anti-CD3 FITC (green-T cells), anti-CD11B FITC (green- macrophages), anti-cytokeratin 19 AF555 (red- bile ducts) and Hoechst nuclear dye (blue).

Based on our observation that Ig-α^-/-^ mice were protected from BA, we sought to determine if the Ig-α^-/-^ mice had changes in the liver immune profile. The BSS Ig-α^-/-^ mice had similar amounts of liver CD4^+^ and CD8^+^ T cells and CD3^+^NKG2D^+^ natural killer (NK) T cells, and small decreases in CD11B^+^macrophages and NKG2D ^+^ NK cells compared to BSS WT mice. The RRV-infected Ig-α^-/-^ mice had significantly decreased numbers of CD4^+^ T cells (P<0.01), CD11B^+^macrophages (P <0.001), NKG2D ^+^ NK cells (P<0.01), and CD3^+^NKG2D ^+^ NK T cells (P<0.001) compared to RRV-infected WT mice (Figure 3A). Immunohistochemistry revealed periductal infiltrates of CD3^+^ T cells and CD11B^+^ macrophages in RRV-infected WT but not Ig-α^-/-^ RRV-infected mice (Figure 3B). These results confirm that RRV-infected Ig-α^-/-^ mice do not develop bile duct-targeted inflammation.

**Figure 4:**
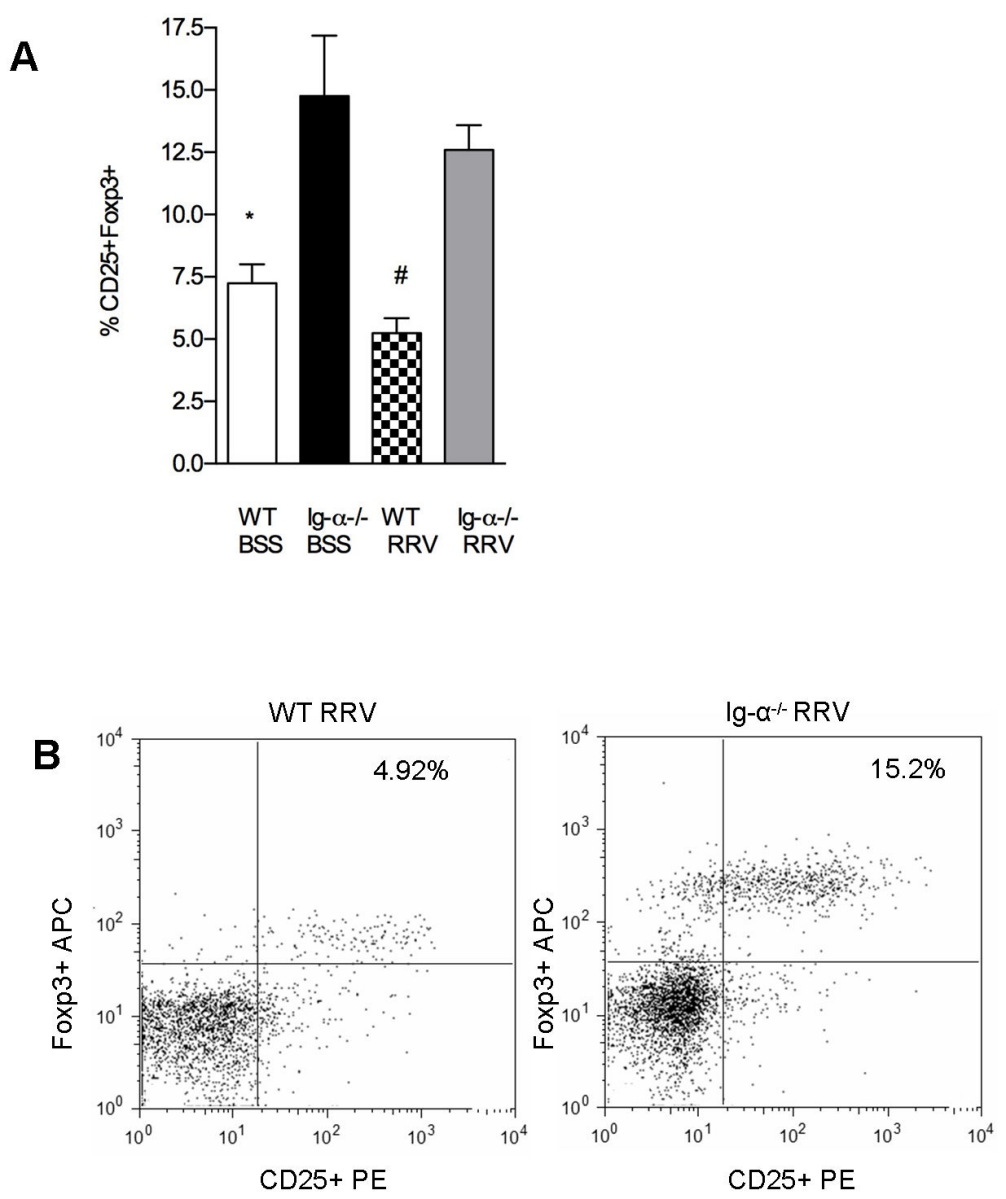
Livers of RRV infected Ig-α ^-/-^ mice have increased regulatory T cells. (A) Summary of liver Treg FACS analysis. Liver immune cells were stained for CD4, CD25 and intranuclear FoxP3. Shown is the percentage of the CD4^+^ population that were CD25^+^Foxp3^+^ (mean ±SD). *P<.001 vs. Ig-α^-/-^ BSS; #P < .001 vs. Ig-α^-/-^ RRV. (B) Representative Tregs dot plots. Shown are representative flow cytometry dot plots of liver Tregs (gated on CD4^+^ cells).

**Figure 5:**
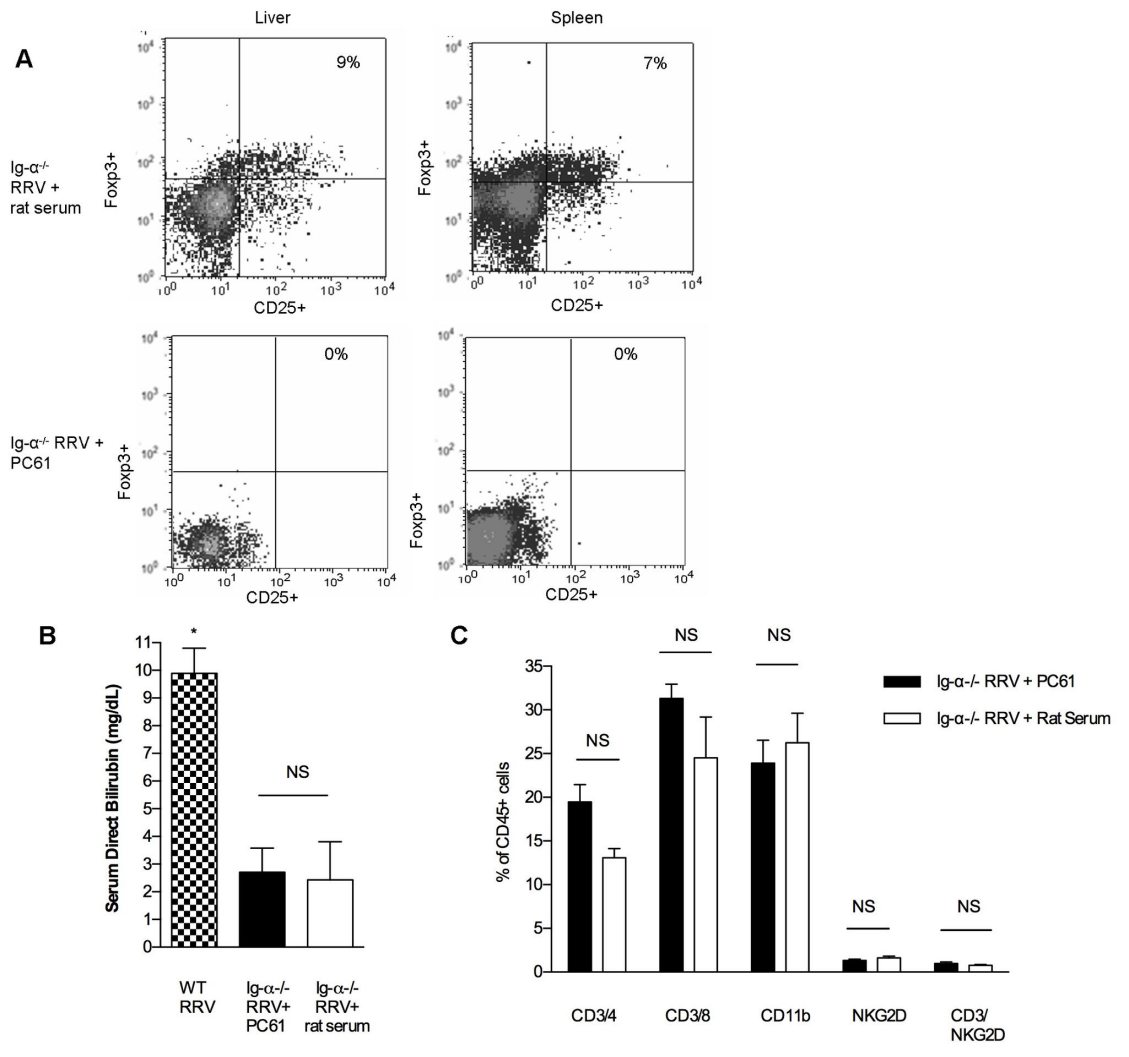
Treg depletion does not induce disease in RRV-infected Ig-α ^-/-^ mice. (A) Successful depletion of Tregs in Ig-α ^-/-^ mice. PC61 (anti-CD25 depleting antibody) was administered to RRV-infected mice on day of life 4. Representative dot plots show PC61 Treg depletion. (B) Serum direct bilirubin (mean ±SD). *P<.001 vs. Ig-α^-/-^ RRV+ PC61 and Ig-α^-/-^ RRV+ rat serum. (C) Liver immune cell profile. Mice were sacrificed at 2 weeks of age and immune cells were identified based on CD45 expression.

Autoimmune diseases are often associated with defects in Treg number and function. Other models of autoimmune disease have identified that B cell depletion results in an increased quantity of Tregs [17,29–31]. Therefore, we sought to determine if Ig-α^-/-^ mice might be protected from disease because of increased levels of Tregs. Increased levels of CD4^+^CD25^+^Foxp3^+^ Tregs were identified in BSS-Ig-α^-/-^ mice (14.76±4.85%) compared to BSS-WT at 2 weeks of age (7.24± 2.56%) (P<0.001). After RRV infection, Tregs remained elevated in the Ig-α^-/-^ mice (12.58± 2.44%), but levels were significantly lower in WT (5.24±1.87%) (P<0.001) (Figure 4A and 4B). To determine if this increased number of Tregs in Ig-α^-/-^ mice was responsible for protection from biliary disease, we performed further experiments utilizing Treg-depleting antibodies (PC61). Neonatal Ig-α^-/-^ mice received RRV at birth, followed by PC61 or rat serum (control) on day 4. Flow cytometry confirmed that mice receiving the PC61 injection were devoid of Tregs (Figure 5A). There was no significant change in disease-free survival between RRV-infected Ig-α^-/-^ mice that received PC61 (79.5%) versus control rat serum (68.5%). Likewise, there was no difference in serum direct bilirubin levels (Figure 5B) or liver immune cell populations (Figure 5C) between RRV-infected Ig-α^-/-^ mice that received PC61 versus control rat serum. Therefore, despite the increase in Tregs in Ig-α^-/-^ mice, these Treg depletion experiments show that Treg cell number was not solely responsible for protection from BA.

### Lack of B cell antigen presentation in Ig-α^-/-^ mice is associated with marked attenuation of CD4^+^ Th1 cell activation

**Figure 6:**
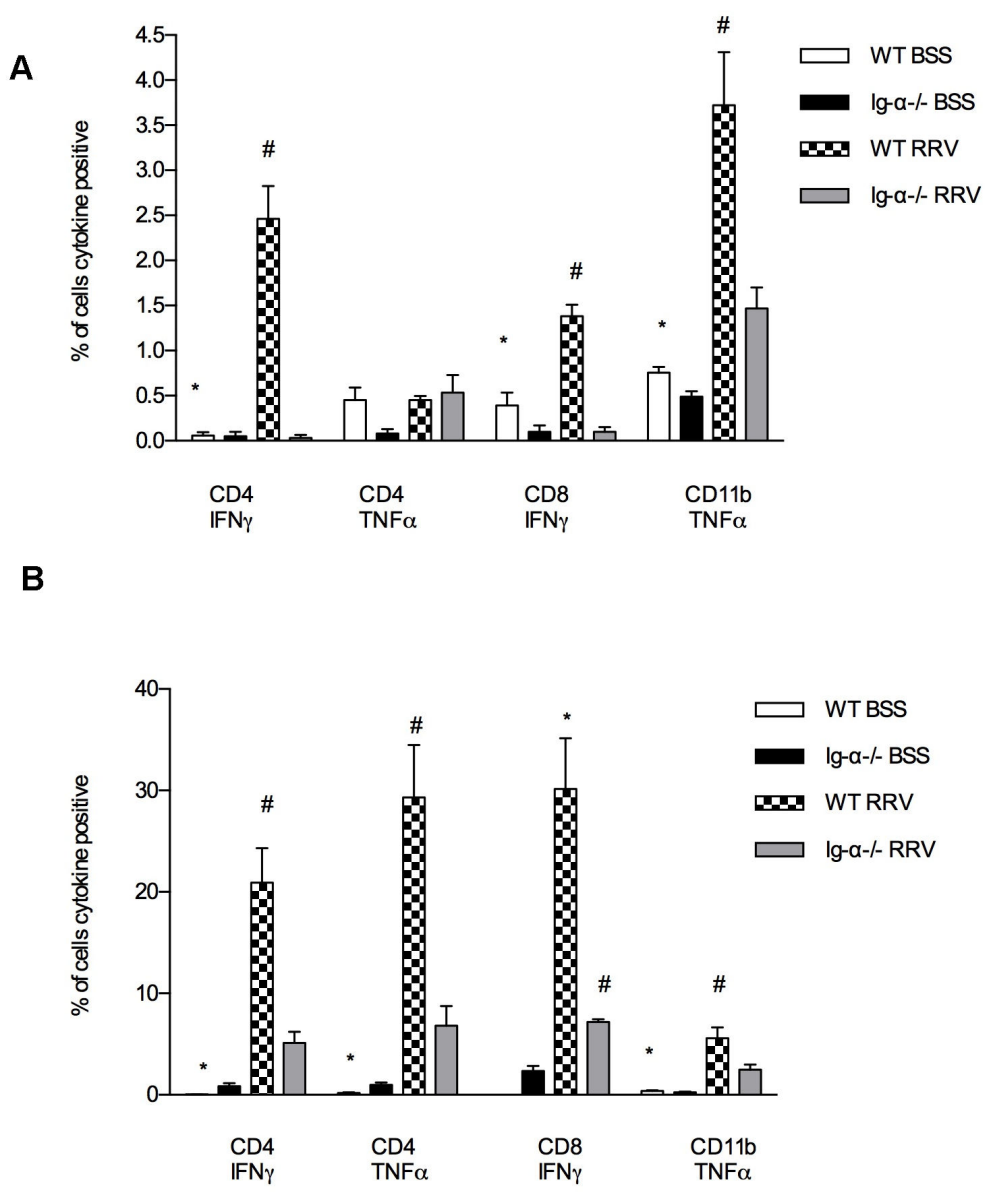
Livers of Ig-α ^-/-^ mice have decreased Th1 cell cytokine production. (A) Ex-vivo baseline Th1 cytokine production. Hepatic immune cells were incubated with Brefeldin A to measure baseline cytokine response from T cells. CD4-IFNγ: *P<.001 vs. WT RRV; #P < .001 vs. Ig-α^-/-^ RRV; CD8-IFNγ: *P<.001 vs. WT RRV; #P < .001 vs. Ig-α^-/-^ RRV; CD11b-TNFα: *P<.001 vs. WT RRV; #P < .01 vs. Ig-α^-/-^ RRV. (B) In vitro stimulation of Th1 cytokine production. Cells were incubated with Brefeldin A followed by stimulation with PMA/ionomycin. CD4-IFNγ: *P<.001 vs. WT RRV; #P < .001 vs. Ig-α^-/-^ RRV; CD4-TNFα: *P<.001 vs. WT RRV; #P < .001 vs. Ig-α^-/-^ RRV; CD8-IFNγ: *P<.001 vs. WT BSS; #P < .001 vs. WT RRV; CD11b-TNFα: *P<.001 vs. WT RRV; #P < .05 vs. Ig-α^-/-^ RRV.

In order for T cells to become activated, they must encounter and recognize antigen displayed on an antigen presenting cell. Professional antigen presenting cells include B cells, macrophages and dendritic cells. To determine if B cell antigen presentation was essential for T cell activation in the mouse model of BA, we assessed the ability of T cells to become activated in the Ig-α ^-/-^ mice. The level of cytokine production from the liver immune cells was determined at baseline (“*ex vivo*”) and after *in vitro* stimulation (PMA/ionomycin). *Ex vivo* analysis reflects the degree of cytokine production at a single time point while *in vitro* stimulation assesses the maximum potential of the immune cell to generate cytokines, reflecting the level of previous immune cell activation. *Ex vivo* analysis demonstrated significantly diminished numbers of CD4^+^ T cells producing IFN-γ (P=0.001), CD8^+^ T cells producing IFN-γ (P=0.001), and CD11B^+^ macrophages generating TNF-α (P=0.01) in RRV-infected Ig-α^-/-^ mice compared to RRV-infected WT (Figure 6A). Liver immune cells from RRV-infected Ig-α^-/-^ mice that were stimulated with PMA/ionomycin *in vitro* also had significantly decreased CD4^+^ T cells producing IFN-γ (P<0.001) and TNF-α (P<0.001), CD8^+^ T cells producing IFN-γ (P=0.001), and CD11B^+^ macrophages producing TNF-α (P<0.05) (Figure 6B). There was no significant difference in production of IL2, IL10 or IL17 in *ex vivo* or *in vitro* analyses (data not shown). These data show that in the absence of B cell antigen presentation, effector T cells (and downstream macrophage activation) from RRV-infected Ig-α^-/-^ mice were markedly less activated, resulting in lack of Th1 inflammatory injury to bile ducts.

## Discussion

**Figure 7:**
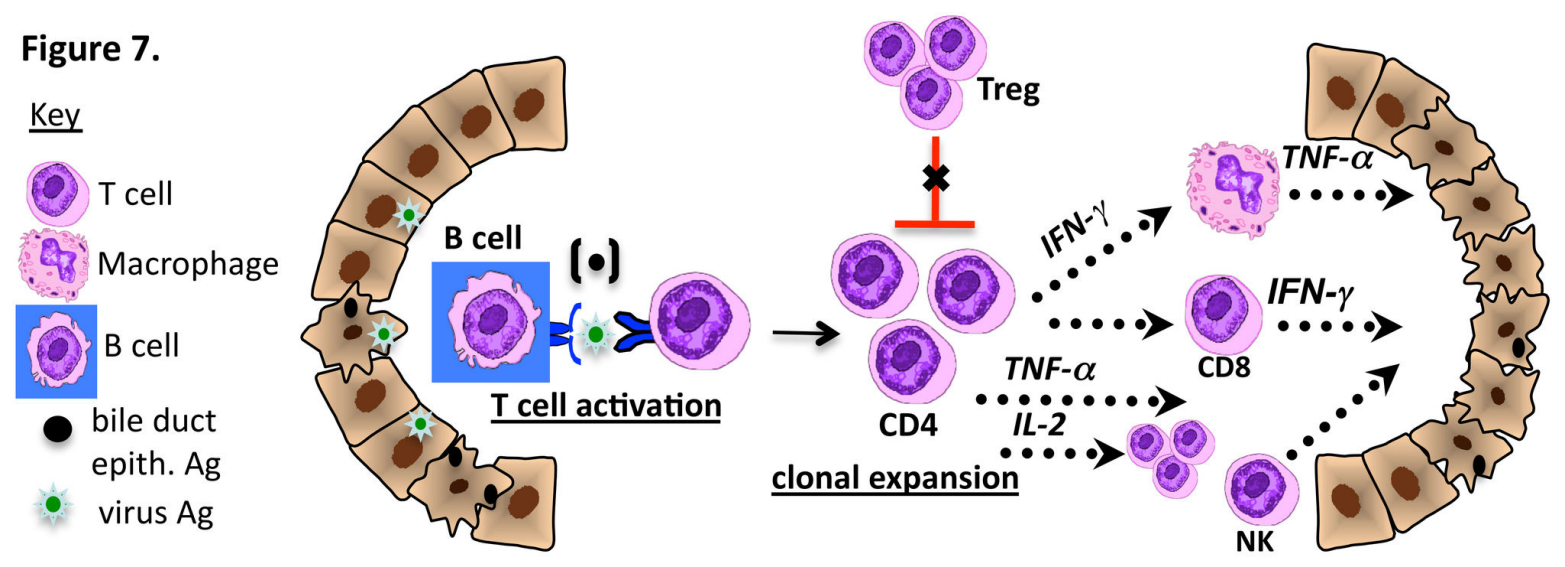
Proposed contribution of B cells to immune-mediated bile duct injury in biliary atresia. Based on the findings from B cell deficient mice, B cells may function as important antigen presenting cells that present virus or bile duct epithelial antigens (Ag) to naïve CD4^+^ T cells with subsequent T cell activation and clonal expansion. Activated Th1 cells: secrete IFN-γ leading to macrophage stimulation; stimulate cytotoxic CD8^+^ T cells; secrete cytotoxic TNF-α and secrete IL-2, leading to further T cell expansion. In addition, B cell activation may inhibit Treg expansion, leading to diminished inhibition of T cell-mediated bile duct injury.

In the present study, we demonstrate that B cell deficient mice are protected from developing BA, suggesting an essential role for B cells in the pathogenesis of RRV-induced BA in mice. The diminished T cell activation in the setting of a B cell-deficient host suggests that the mechanism of disease protection involves lack of B cell antigen presentation. A summary of the proposed mechanism of action of B cells in murine BA is shown in Figure 7. Limitations to this study include the fact that macrophage numbers were mildly decreased in the Ig-α^-/-^ mice, possibly contributing to diminished antigen presentation. In addition, B cells secrete lymphotoxin which is required for proper lymphoid development and, together with other B cell factors, regulates dendritic cell and T cell interactions [32]. Saxena et al. has demonstrated that primed dendritic cells are required for the proliferation of T lymphocytes and the activation of NK cells in experimental BA [33].

B cells have been shown to be critical in the development and maintenance of multiple autoimmune diseases [17,19,20,34,35]. In a recent study by Chan et al. [36], mice unable to secrete immunoglobulin still developed lupus nephritis, suggesting that the role of B cells in autoimmune nephritis extends beyond autoantibody production and includes antigen presentation function. B cells are unique in their ability to stimulate autoimmune responses. Unlike other professional antigen cells which recognize molecular patterns from an antigen as foreign, the B cell receptor cannot distinguish between self and foreign antigens. Therefore, they are able to present self antigens to CD4 T cells and initiate an autoreactive immune response. Additionally, the B cell receptor’s high affinity for a specific antigen allows for efficient processing of even small amounts of antigen that escape sequestration from the immune system. Finally, the processing of autoantigen by B cells is required for epitope spreading, which results in augmentation of an autoimmune response [37]. The essential role of antigen-specific B cells as antigen presenting cells has been demonstrated in the mouse models of systemic lupus erythematosus [38], diabetes [20,39,40], and autoimmune arthritis [19] where B cells are necessary for the activation of autoreactive T cells. In the animal model of multiple sclerosis, non-B antigen presenting cells can process and present antigenic protein, but are not able to induce the same pathogenic immune response initiated when B cells present antigen [41]. Several murine experimental models have demonstrated that B cell deficiency results in failure to prime CD4^+^ T cells [42,43]. Autoantigen-specific B cells have been shown to improve Th1 [44,45], Th2 [46], and memory responses [47]. In addition, B cells have been shown to express a number of co-stimulatory molecules which reinforce priming and reactivation of antigen-specific T cells [48,49]. The findings of this study corroborate the important role of B cells in inflammatory mediated bile duct injury in the RRV mouse model of BA.

Tregs function to suppress inflammation as well as autoreactive T cells that escape into the periphery. Quantitative and qualitative reduction of Tregs has been demonstrated in multiple inflammatory and autoimmune diseases [50–52], including both the mouse model and human studies of BA [53–55]. A link between B cells and Treg activity has been suggested in the mouse model of Crohn’s disease where B cells worsen ileitis through suppression of Treg function [56]. Interestingly, in multiple animal models as well as clinical studies of other autoimmune diseases, B cell depletion has resulted in an increased quantity and improved suppressive abilities of Tregs [17,29–31]. In this study, we identified increased quantity of Tregs at baseline and after RRV infection in B cell deficient mice. We initially hypothesized that this increased quantity of Tregs may have contributed to protection from disease. Although depletion of Tregs did not induce disease in Ig-α^-/-^ mice treated with PC61, these mice did develop slightly higher serum bilirubin levels (3 mg/dl compared to <1 mg/dL) suggesting some degree of biliary injury, though not enough to lead to complete biliary obstruction. Therefore, while increased quantity of Tregs in Ig-α^-/-^ mice was not solely responsible for protection from disease, it may play a minor role.

This is the first study to demonstrate that B cell deficient mice are protected against bile duct injury and obstruction in the RRV-induced mouse model of BA. These findings have important clinical implications. If B cells are required to activate CD4^+^ T cells and perpetuate ongoing inflammation and bile duct injury, then strategies to deplete or inhibit B cell function might ameliorate bile duct targeted inflammation and could potentially delay or prevent the need for liver transplantation in children with BA. B cell directed therapies have yielded promising results in cohorts of patients with rheumatoid arthritis [57], multiple sclerosis [58,59], and type 1 diabetes mellitus [60] confirming the important role of B cells in pathogenesis of human autoimmune disease. Further exploration of the mechanisms by which B-cell depletion may be protective in human BA are indicated.
